# A 27-year-old Female Patient with Acute nausea/vomiting and Pelvic pain; a Photo Quiz

**DOI:** 10.22037/aaem.v10i1.1508

**Published:** 2022-04-24

**Authors:** Murat Ozsarac, Yusuf Yurumez, Onur Karakayali

**Affiliations:** 1 Department of Emergency Medicine, Sakarya University Faculty of Medicine, Sakarya, Turkey.

**Keywords:** Pelvic pain, ovary, varicose veins, emergency medicine

## 1. Case presentation:

**Figure 1:**
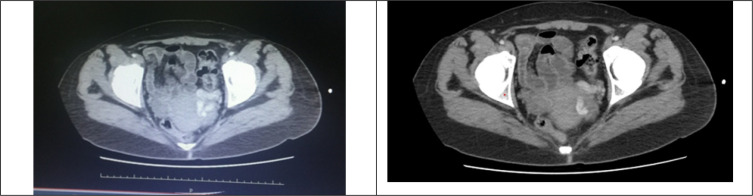
Axial view of intravenous contrast-enhanced abdominopelvic computed tomography scan of the patient

A 27-year-old female patient, G2P1, presented to the emergency department (ED) with acute onset nausea, vomiting, and mild chronic abdominopelvic pain. Physical examination revealed bilateral lower quadrant tenderness without rebound, guarding, or rigidity, and vital signs were within normal limits. Electrolytes, complete blood count, and liver and kidney function tests were normal. A pregnancy test was negative, and urinalysis did not reveal any abnormalities. No free fluid was observed in the abdominal ultrasound, and the ovaries and other intra-abdominal structures were found to be normal. The patient underwent intravenous contrast-enhanced abdomiopelvic computed tomography (CT) scan (figure 1).

## 2. Diagnosis:

**Figure 2:**
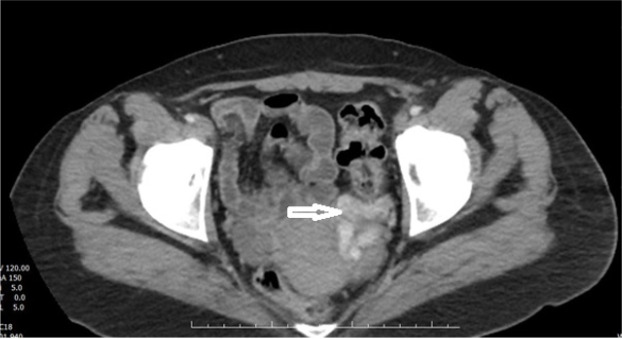
Axial view of intravenus contrast-enhanced abdominopelvic computed tomography scan demonstrates dilatation in the left gonadal vein and dilated vascular structures (white arrow).

Intravenous contrast-enhanced abdominopelviccomputed tomography (CT) scan shows dilatation in the left gonadal vein and dilated vascular structures in the left parauterine area (figure 2) in favor of pelvic congestion syndrome (PCS).

The differential diagnosis of pelvic pain is broad. Many etiological causes related to gynecological, gastrointestinal, urinary, vascular, nervous, and musculoskeletal systems should be considered (1). Pelvic Congestion Syndrome (PCS) might be one of the most common, underdiagnosed causes of pelvic pain in female patients. It is also an entity not sufficiently recognized in emergency medicine practice. The etiology of congestion is quite complex due to hormonal and structural causes. Valvular insufficiency, reflux, and venous obstruction play an important role in developing congestion and stasis (2). This text presents and discusses a PCS case who presented to the Emergency Department (ED) with abdominopelvic pain and nausea/vomiting.

## 3. Case fate:

Antiemetics and analgesics were administered. No intervention was performed for the patient after consultation with obstetrics and gynecology. A follow-up by the cardiovascular surgery department was planned. The control trans-vaginal ultrasound examination performed at the outpatient clinic two weeks later was evaluated as normal. No malignancy or intraepithelial lesion was observed in the cervical smear. Analgesics were recommended to the patient, and symptom follow-up was planned.

## 4. Discussion:

The diagnosis of PCS does not provide a clue to to understand the underlying mechanism of disorders of the pelvic venous circulations. The clinical manifestations in PCS are chronic pelvic pain lasting longer than six months, aggravated by prolonged standing, intercourse, and menstruation, as well as lower back pain, urinary symptoms such as dysuria, urgency, frequent urination, and vaginal discharge (3). It typically affects young multiparous women between the ages of 20 and 30, although it can rarely be seen in pregnant women and during the postmenopausal period (4). Pelvic varices are accompanied by lower extremity varices and chronic venous insufficiency in 10%-70% of cases. It is less frequently associated with the enlargement of thigh, perineal, vulvar and saphenous veins. Particularly, the development of edema in the legs, varicose veins of the lower extremities, pain, and heaviness in the legs are directly related to the reflux that develops in common anatomical connections (5). As a non-invasive screening tool, Doppler ultrasound is a reasonable initial option as it allows real-time dynamic imaging and flow evaluation. Transvaginal ultrasound and Doppler ultrasound criteria for PCS diagnosis include a dilated, parauterine, and paraovarian vein greater than 4 mm in diameter or retrograde flow (1, 4). Color Doppler in the upright position with Valsalva manuver significantly increases the diagnostic accuracy (1). PCS imaging criteria for CT and magnetic resonance imaging (MRI) include the presence of at least four ipsilateral coiled parauterine vessels or an ovarian vein diameter greater than 8 mm (4). CT and MRI are better diagnostic tools than ultrasound as they provide a detailed anatomical examination of dilated pelvic and ovarian veins. However, CT and MRI cross-sectional images obtained in supine position play a limited role in diagnosing PCS and detecting dilatation of the thin pelvic veins (6). Once the diagnosis is confirmed, PCS management includes medical, surgical, and endovascular approaches. Different treatment modalities have been tried for embolization of abnormal pelvic veins, providing symptom relief in 75% of patients (5). 

## 5. Conclusion:

Contrast-enhanced CT scan may provide an incidental diagnosis of PCS in patients with frequent presentations due to pelvic pain. However, the diagnosis can be easily overlooked unless Doppler ultrasonography is performed in the semi-supine or upright position. Emergency physicians must recognize this common but overlooked clinical condition.

## 6. Declarations:

### 6.1. Acknowledgements

We would like to thank Lingus and BSB group for English language editing.

### 6.2. Authors’ relationships

All authors met the criteria for authorship contribution based on the international committee of medical journal editors’ recommendations.

### 6.3. Conflict of interest

The authors declare no conflicts of interest.

### 6.4. Funding

None

### 6.5. Informed consent for publication

The photo quiz was written in an anonymous characteristic, thus confidential and detailed data about the patient is removed. Editor and reviewers can know and see these detailed data.
